# CRISPR/Cas9-mediated mutagenesis of *VvMLO3* results in enhanced resistance to powdery mildew in grapevine (*Vitis vinifera*)

**DOI:** 10.1038/s41438-020-0339-8

**Published:** 2020-08-01

**Authors:** Dong-Yan Wan, Ye Guo, Yuan Cheng, Yang Hu, Shunyuan Xiao, Yuejin Wang, Ying-Qiang Wen

**Affiliations:** 1 State Key Laboratory of Crop Stress Biology for Arid Areas, College of Horticulture, Northwest A&F University, Yangling, 712100 Shaanxi China; 2 Key Laboratory of Horticultural Plant Biology and Germplasm Innovation in Northwest China, Ministry of Agriculture, Yangling, 712100 Shaanxi China; 3 Institute for Bioscience and Biotechnology Research & Department of Plant Sciences and Landscape Architecture, University of Maryland College Park, Rockville, MD 20850 USA

**Keywords:** DNA damage and repair, Mutagenesis, Plant breeding

## Abstract

Grapevine (*Vitis vinifera*), one of the most economically important fruit crops in the world, suffers significant yield losses from powdery mildew, a major fungal disease caused by *Erysiphe necator*. In addition to suppressing host immunity, phytopathogens modulate host proteins termed susceptibility (S) factors to promote their proliferation in plants. In this study, CRISPR/Cas9 (clustered regularly interspaced short palindromic repeats/CRISPR-associated 9) technology was used to enable the targeted mutagenesis of *MLO* (mildew resistance Locus O) family genes that are thought to serve as *S* factors for powdery mildew fungi. Small deletions or insertions were induced in one or both alleles of two grapevine *MLO* genes, *VvMLO3* and *VvMLO4*, in the transgenic plantlets of the powdery mildew-susceptible cultivar Thompson Seedless. The editing efficiency achieved with different CRISPR/Cas9 constructs varied from 0 to 38.5%. Among the 20 *VvMLO3/4*-edited lines obtained, one was homozygous for a single mutation, three harbored biallelic mutations, seven were heterozygous for the mutations, and nine were chimeric, as indicated by the presence of more than two mutated alleles in each line. Six of the 20 *VvMLO3/4*-edited grapevine lines showed normal growth, while the remaining lines exhibited senescence-like chlorosis and necrosis. Importantly, four *VvMLO3*-edited lines showed enhanced resistance to powdery mildew, which was associated with host cell death, cell wall apposition (CWA) and H_2_O_2_ accumulation. Taken together, our results demonstrate that CRISPR/Cas9 genome-editing technology can be successfully used to induce targeted mutations in genes of interest to improve traits of economic importance, such as disease resistance in grapevines.

## Introduction

Grapevine is one of the most extensively cultivated and economically valuable horticultural crops in the world, with 7.6 million hectares in production and an annual value of ~$3.6 billion in wine export markets alone (Organisation Internationale de la Vigne et du Vin, 2017). Unfortunately, grape production is limited by various diseases, including powdery mildew caused by the fungus *Erysiphe necator*. Powdery mildew can be controlled to a certain extent by fungicides, but this increases production costs and can negatively affect the environment and human health. In addition, the indiscriminate use of pesticides increases carbon emissions^1^.

A more cost-effective and environmentally friendly strategy for limiting losses due to diseases is the development of disease-resistant cultivars through traditional breeding and/or precision breeding with the aid of biotechnology. The identification and characterization of the genes and genetic pathways that confer resistance can greatly facilitate the latter approach. Advances in functional genomics are changing the preferred experimental strategy from using traditional forward genetics (i.e., from phenotype to genotype) to employing both forward genetics and reverse genetics (i.e., inferring gene function based on the phenotypes caused by mutations of a target gene)^2^. The introduction of functional mutations through targeted genome editing is a powerful reverse genetics approach for understanding gene function. CRISPR-Cas9 technology is a relatively simple yet effective technique that can be exploited for gene mutation, the repression/activation of gene expression, subtle modification of gene function and epigenome editing^3^. In 2013, three research groups simultaneously reported the use of the CRISPR-Cas9 system for targeted genome modification in *Arabidopsis thaliana* (Arabidopsis), tobacco, rice and wheat for the first time^4–6^. This system has also been successfully applied in maize^7^, cotton^8^, and other plants. Grapevine is a woody perennial plant with a highly heterozygous genome that can be genetically transformed only through lengthy and difficult procedures. To date, there have been only a few reports on the use of CRISPR-Cas9 for genome editing in grapevine^5,9–11^.

Over the past 25 years, the plant immune system has been extensively studied, and fundamental discoveries have led to a better understanding of the molecular basis of plant disease resistance. While the development of disease-resistant crop varieties has typically relied on the introduction of dominant resistance (*R*) genes into elite cultivars via classical breeding, recessive mutations in host susceptibility (*S*) genes have also been utilized to confer resistance. *R*-gene-induced defense responses often result in localized cell death or a hypersensitive response (HR)^12^; by contrast, the loss of *S*-genes required for the successful invasion of a particular pathogen may not display strong defense activation. For example, the disruption of *S*-genes such as *PMR5*^13^, *DMR6*^14,15^, *LOB1*^16^, and *SWEET14*^17,18^ can confer broad-spectrum disease resistance with no or minimal activation of defenses in several economically important plant species. *S*-gene-mediated resistance is generally more durable than *R*-gene-based resistance because the pathogen must overcome dependence on a host S factor^15^. The disruption of the *MLO* gene in barley confers durable powdery mildew resistance, as demonstrated by the fact that no powdery mildew strains capable of overcoming *mlo*-mediated resistance have been found in the field for many decades^19^.

Studies suggest that the pathogenicity of adapted powdery mildew species relies on a functional host *MLO* gene^20,21^. MLO proteins constitute a family of highly conserved^22^, plant-specific, seven-transmembrane domain proteins that are structurally similar to G protein-coupled receptors (GPCRs) with a calmodulin-binding domain^23^. The mechanism by which MLO proteins act as powdery mildew susceptibility factors is unknown, and the biochemical function of MLO proteins in the host remains largely unexplored^24^. In barley, an *mlo* mutation was found to confer broad-spectrum and durable resistance to barley powdery mildew isolates of *Blumeria graminis* f. sp. *hordei*^25^, but a range of developmentally controlled pleiotropic effects occurred in the host, such as the formation of callose-containing cell wall appositions (papillae) in the absence of any pathogen and the premature onset of leaf senescence. These effects reduced the grain yield of the *mlo* mutants; thus, the dysfunctional MLO proteins may indeed result in a competitive disadvantage in natural populations^26^. In bread wheat, TALEN-induced mutations in all three *TaMLO* homologs confer heritable broad-spectrum resistance to powdery mildew^27^. In apple, the suppression of *MdMLO11* and *MdMLO19* through RNA interference (RNAi) reduces susceptibility to powdery mildew (*Podosphaera leucotricha*)^28^. In *Arabidopsis*, the *Atmlo2 Atmlo6 Atmlo12* (*Atmlo2*/*6*/*12*) triple mutant is completely resistant to powdery mildew^21^. The grapevine genes *VvMLO3*, *VvMLO4*, and *VvMLO17*, orthologous to the *AtMLO2*, *AtMLO6*, and *AtMLO12* genes, respectively^29^, are reported to exhibit rapid induction upon powdery mildew infection, suggesting that these VvMLO proteins may play a role in modulating antifungal defense responses in grapevine. One previous study used RNAi to suppress specific *VvMLO* genes and found that the simultaneous knockdown of *VvMLO6* and *VvMLO7* in grapevine reduced susceptibility to powdery mildew^30^.

The grapevine cultivar Thompson Seedless is an ideal material for studying powdery mildew resistance in grapevine because it is amenable to transformation and susceptible to powdery mildew infection. There are 17 *VvMLO*s in Thompson Seedless. In this study, we used CRISPR/Cas9 technology to target two grapevine *MLO* genes, *VvMLO3* and *VvMLO4*, and obtained *VvMLO3*-edited grapevine lines with enhanced resistance to powdery mildew. Our work suggests that CRISPR/Cas9-targeted mutagenesis is a valuable tool for generating disease-resistant grapevines.

## Results

### Target selection within *MLO*s and vector construction

**Fig. 1:**
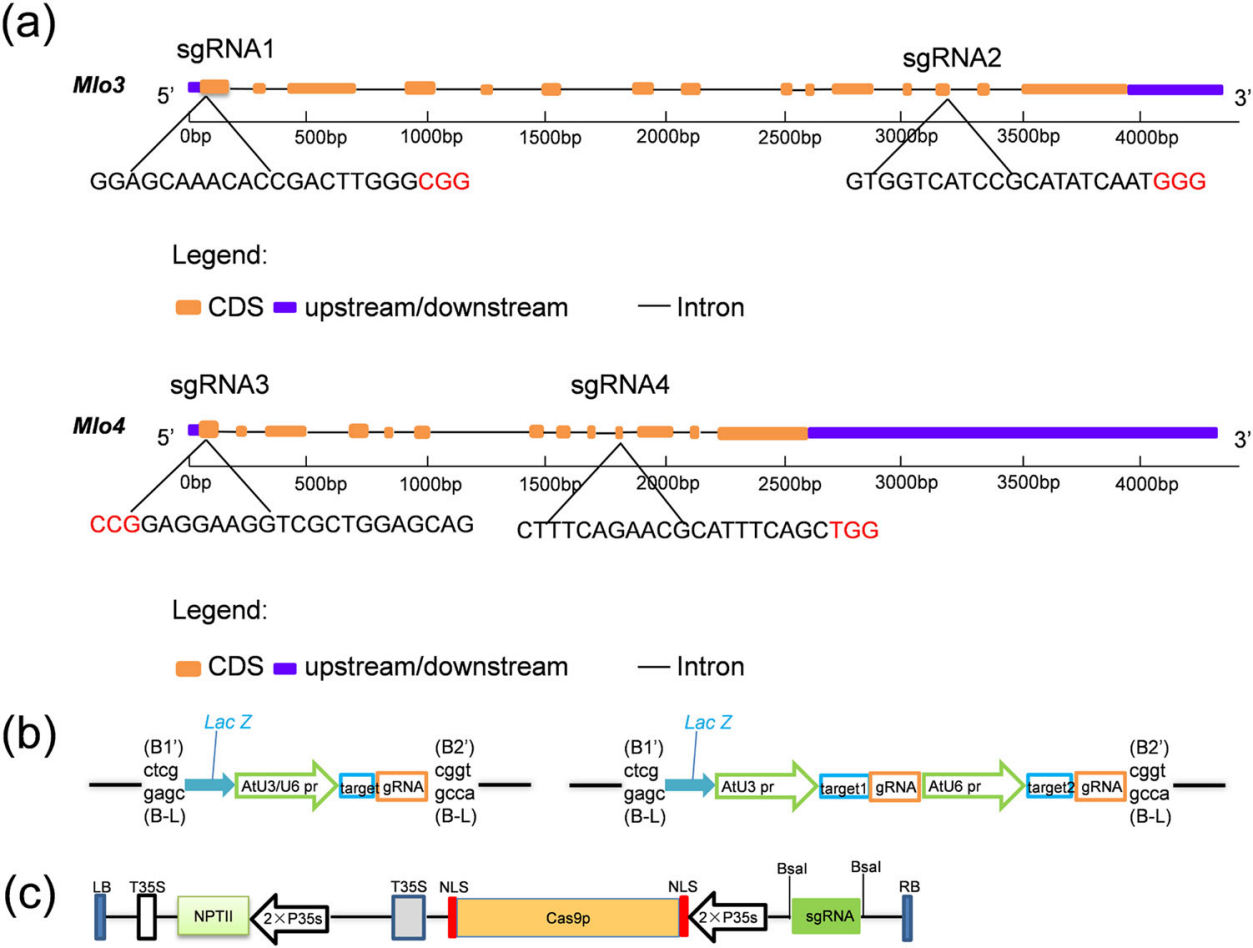
Target selection and expression constructs for Cas9 and sgRNA. **a** Target selection of *VvMLO3* and *VvMLO4*. CRISPR/Cas9-targeted sites were selected within exons 1 (sgRNA1) and 13 (sgRNA2) of *VvMLO3* and exons 1 (sgRNA3) and 10 (sgRNA4) of *VvMLO4*. The protospacer-adjacent motif (PAM) sequence is highlighted in red. **b**, **c** Illustration of the assembly of the pYLCRISPR/Cas9 binary vector combined with the sgRNA by using Golden Gate cloning.

For each *VvMLO* gene, two target loci were selected (Fig. 1a). Schematic representations of the target sites of two small guide (sg) RNAs and their gene expression cassettes used for the *Agrobacterium*-mediated transformation of grapevine embryogenic calli is shown in Fig. 1a, b. The sgRNA intermediate vectors are based on the pUC18 backbone described previously^31^. The binary vector for plant transformation contains a plant codon-optimized *Cas9* gene. In total, four DNA constructs, designated CM3G1 (**C**as9/sgRNA**1**-***M****LO3*-**G**rapevine), CM3G2, and CM4G3 (**C**as9/sgRNA**3**-***M****LO4*-**G**rapevine) and CM4G4, were produced and used for transformation (Fig. 1b, c).

### Targeted mutagenesis in regenerated transgenic grapevines

**Fig. 2:**
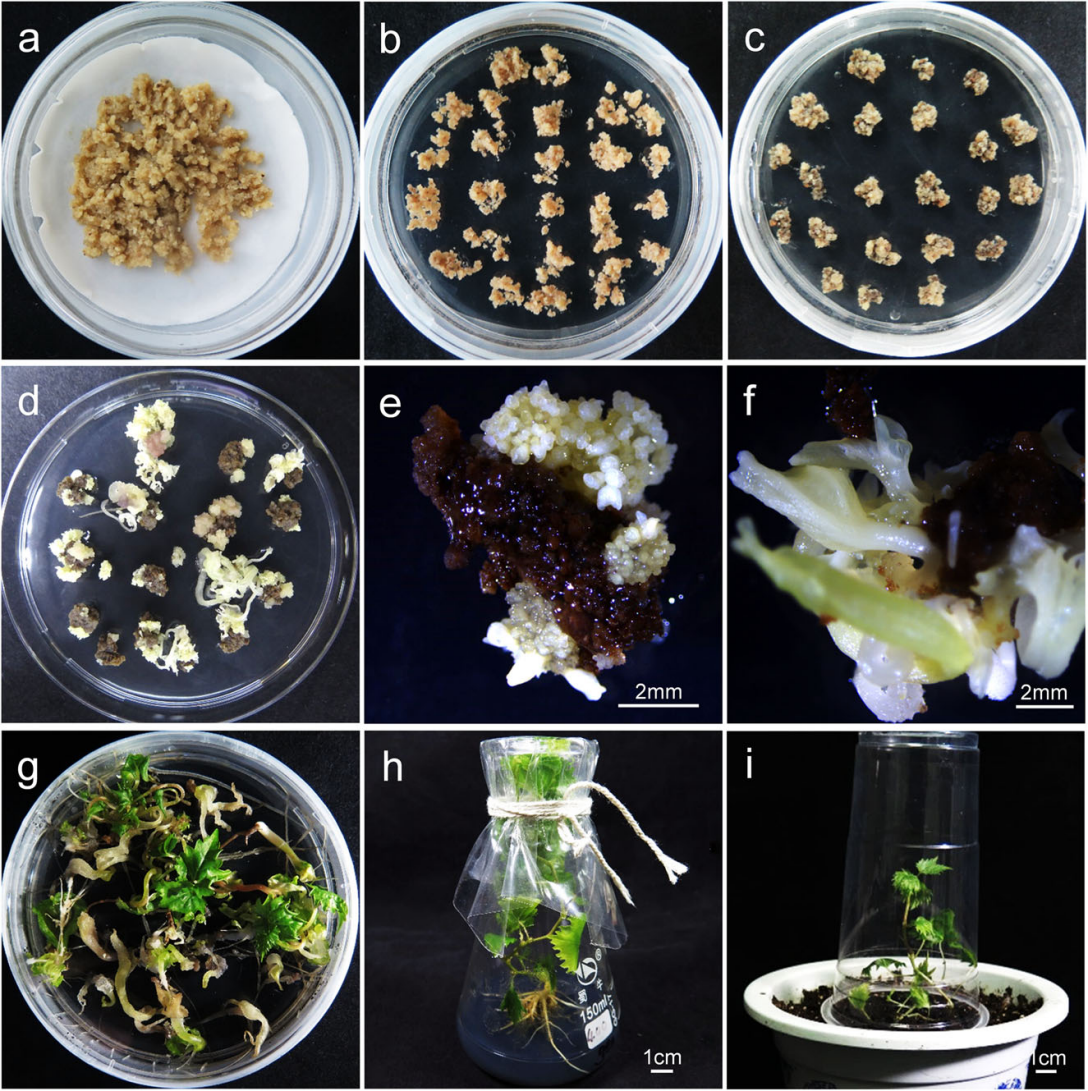
Genetic transformation of *Vitis vinifera* cv. Thompson Seedless by *Agrobacterium tumefaciens* and the regeneration of transgenic lines. **a** Coculture of proembryonic masses (PEMs) and *Agrobacterium tumefaciens* on two layers of filter paper soaked in 2 ml suspension liquid (MS + 200 μmol AS). **b** The transformed PEMs cultured on X3CC (X3 + 200 mg·L^−1^ carb + 200 mg·L^−1^ Cef) medium after the elimination of the bacteria. **c** The transformed PEMs cultured on X3CCK75 (X3 + 200 mg·L^−1^ carb + 200 mg·L^−1^ Cef + 75 mg·L^−1^ kanamycin) medium. **d**–**f** Appearance of kanamycin-resistant somatic embryos. **g** Kanamycin-resistant somatic embryos transferred to germination medium and cultured under light. **h** Germination of resistant plantlets. **i***MLO*-edited lines domesticated in the phytotron.

**Fig. 3:**
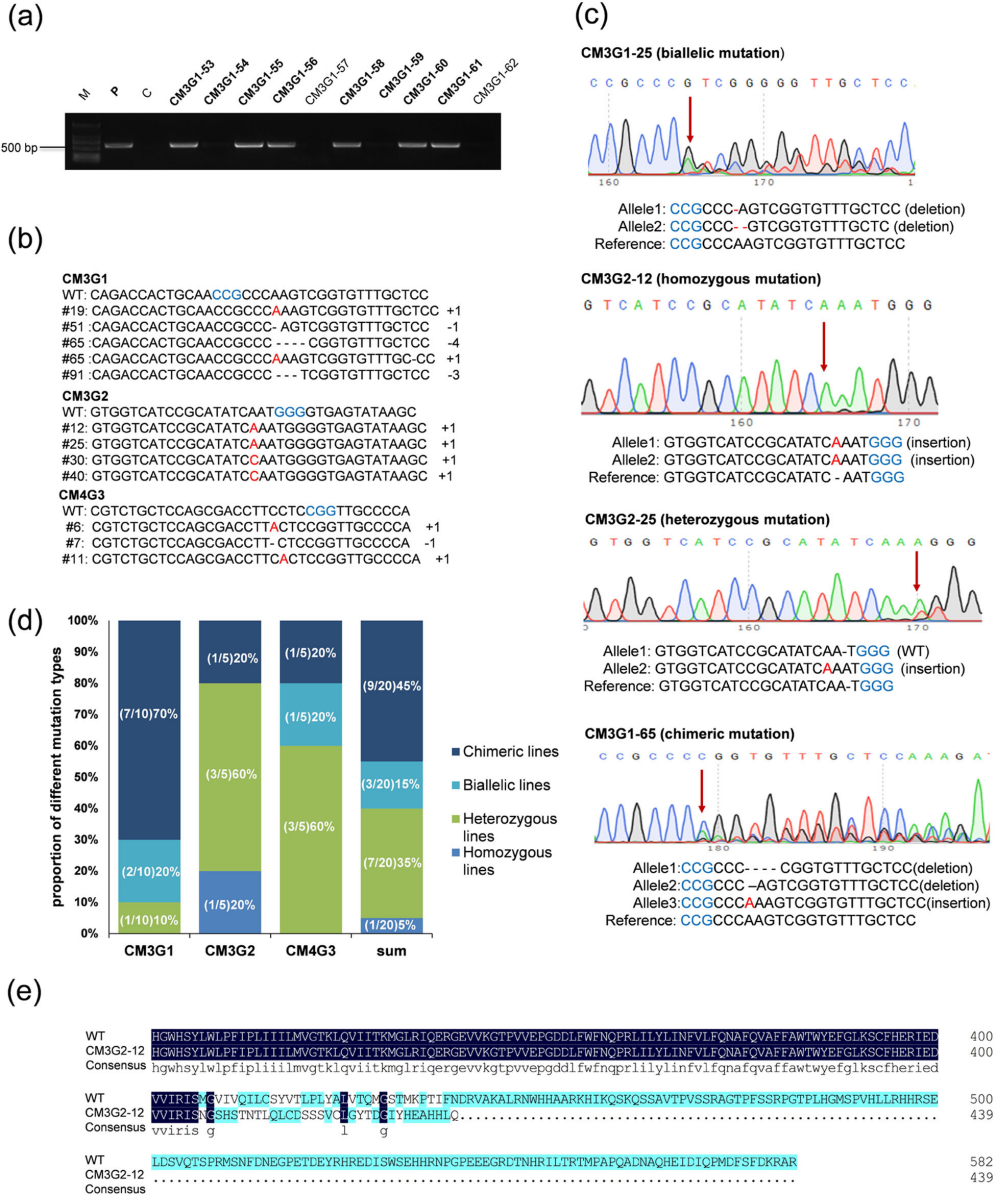
Targeted mutagenesis of *VvMLO3* and *VvMLO4* using the CRISPR-Cas9 system. **a** PCR amplification of a 507-bp DNA fragment of genomic DNA from regenerated plantlets using gene-specific primers. M: marker, P: plasmid, C: control, CM3G1-53 to CM3G1-62: different regenerated lines expressing *VvMLO3*-sgRNA1. **b** The numbers on the right indicate the type of mutation and the number of nucleotides involved, “−” and “+” indicate deletions and insertions, respectively. The insertions are highlighted in red letters, and the protospacer-adjacent motif (PAM) is indicated in blue letters. **c** Four different types of mutation sequencing chromatograms: biallelic, homozygous, heterozygous, and chimeric mutations. The first substitution or indel sites are indicated with red arrowheads. The protospacer-adjacent motif (PAM) sequences are highlighted in blue, the indels or substituted bases are highlighted in red, and ‘−’ indicates deletions. **d** The frequency of different mutation types found in the edited lines. **e** Amino acid sequence alignment between one representative mutant and the wild-type for the target region of VvMLO3.

In total, 210 plantlets were regenerated from the grapevine anther filament-induced embryogenic calli subjected to coincubation with *Agrobacterium* cells (Fig. 2). To verify the presence of the CRISPR-Cas9 sequence, genomic DNA from each regenerated plantlet was analyzed by PCR. As shown in Fig. 3a, a fragment of ~508 bp was amplified from *Cas9*-positive plantlets, whereas *Cas9*-negative and nontransgenic control plantlets showed no amplification. A total of 125 plantlets were identified as *Cas9* positive for any of the four targets of the two *VvMLO* genes, and these plantlets were further analyzed for the presence of the intended sequence variation. A total of 68 independent PCR-positive plantlets for *VvMLO3*-sgRNA1, 39 for *VvMLO3*-sgRNA2, 13 for *VvMLO4*-sgRNA3, and 5 for *VvMLO4*-sgRNA4 were analyzed by Sanger sequencing (Table 1). The corresponding DNA target sites of sgRNA1, sgRNA2, or sgRNA3 contained mutations (Table 1), indicating that three of the four CRISPR/Cas9-DNA constructs (i.e., CM3G1, CM3G2, CM4G3) enabled successful gene editing in the regenerated grapevine plantlets. Different target sites were mutated with different editing efficiencies, varying from 0 to 38.5% (Table 1). Single-nucleotide insertions and nucleotide deletions of various lengths were identified among these edited lines (Fig. 3b). The mutations detected in the sgRNA1 lines were mainly small deletions and insertions, while those of the sgRNA2 lines were mainly small insertions, and those of the sgRNA3 lines were mainly small insertions (Fig. 3b and Table S1). Most of the insertions consisted of only 1 bp, and the majority of deletions were short in the sgRNA1 and sgRNA2 lines. Double-strand breaks (DSBs) usually occur at a position three base pairs upstream of the PAM sequence^32^. Among the 20 *VvMLO*-edited lines, 16 showed mutation sites exactly at the 4th base from the PAM site (Fig. S1 and Table S1).

**Table 1:** Summary of genome editing results in regenerated plantlets.

	CM3G1	CM3G2	CM4G3	CM4G4
Regenerated lines	117	63	22	8
Cas9 positive lines	68	39	13	5
Homozygous^a^ lines	0	1	0	0
Heterozygous^b^ lines	1	3	3	0
Biallelic^c^ lines	2	0	1	0
Chimeric^d^ lines	7	1	1	0
Nonedited lines	107	58	17	8
Editing efficiency	14.7%	12.8%	38.5%	0

^a^Homozygous = both alleles contain the same mutation.

^b^Heterozygous = only one allele is mutated.

^c^Biallelic = both alleles are mutated, but the mutations are not identical.

^d^Chimeric = presence of more than two mutant alleles, indicative of the presence of different edited cell lines.

Four different types of mutation sequence chromatograms are shown in Fig. 3c. These 20 edited lines were detected among the 125 transgenic plantlets that were obtained and analyzed (Table 1); thus, the overall gene-editing efficiency was 16.5%. It was interesting that no edited lines were detected among any of the five transgenic lines containing the CM4G4 construct. Among the 20 edited lines, one (5%) was homozygous for a 1-bp insertion, three (15%) were biallelic for small deletions and insertions (Indels), seven (35%) were heterozygous (i.e., only one allele contained a mutation), and nine (45%) were chimeric (i.e., more than two mutated alleles were detected in one line) (Fig. 3d). Detailed information about the mutation found in one edited line, CM3G2-12, is provided as an example in Fig. 3e; both alleles of *VvMLO3* in this line contained a single-nucleotide (A) insertion in the target site, resulting in the formation of a premature stop codon, which in turn was predicted to produce a VvMLO3 mutant with a C-terminal truncation.

### Phenotypic characterization of genome-edited lines

**Fig. 4:**
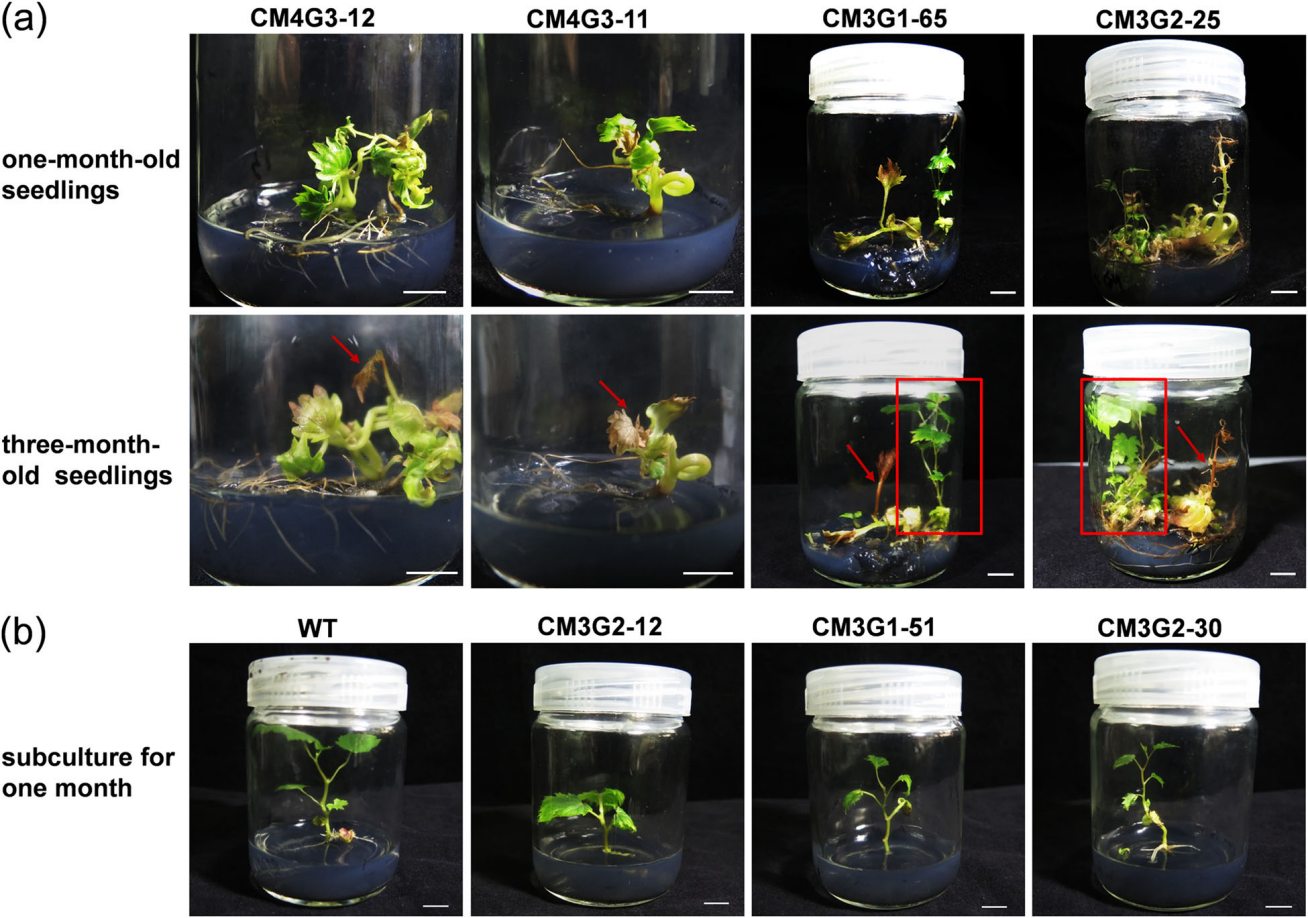
*VvMLO* mutant grapevine plantlets showed early senescence-like phenotypes and difficulty in rooting. **a** Targeted *VvMLO*-edited plantlets showed early senescence-like phenotypes. A: 1-month-old *VvMLO*-edited plantlets grown in the rooting medium, B: 3-month-old *VvMLO*-edited plantlets grown in the rooting medium. Red arrows indicate chlorosis, and red box frames indicate the regenerated nonedited plantlets. Bars = 1 cm. **b** Rooting of the indicated *VvMLO*-edited and nonedited wild-type (WT) plantlets in rooting medium. Bars = 1 cm.

The vast majority (93%) of the regenerants obtained after *Agrobacterium* infection in this study showed abnormal growth and development (not shown). When grown under axenic conditions, 14 of 20 3-month-old *VvMLO3-* or *VvMLO4*-edited plantlets (e.g., CM3G1-65 and CM3G2-25; Fig. 4a) showed similar phenotypes, which were characterized by senescence-like chlorosis and necrosis. Among these 14 plantlets, CM3G2-12, homozygous for a 1-bp insertion, died in the medium when it was two months old. However, the nonedited plants displayed normal growth and development during the same period.

We found that when the *VvMLO*-edited plantlets were subcultured to induce proliferation, their rooting potential was very low, even in the presence of IBA at a concentration as high as 1.0 mg·L^−1^. Only a few lines (i.e., CM3G2-30 and CM3G1-51) showed normal rooting (Fig. 4b). Compared with the nontransgenic control, the growth of the *VvMLO*-edited plantlets was poor, and the lower part of the plantlets showed accelerated defoliation.

### Powdery mildew-triggered mesophyll cell death, cell wall apposition and H_2_O_2_ production are associated with *mlo*-mediated resistance

**Fig. 5:**
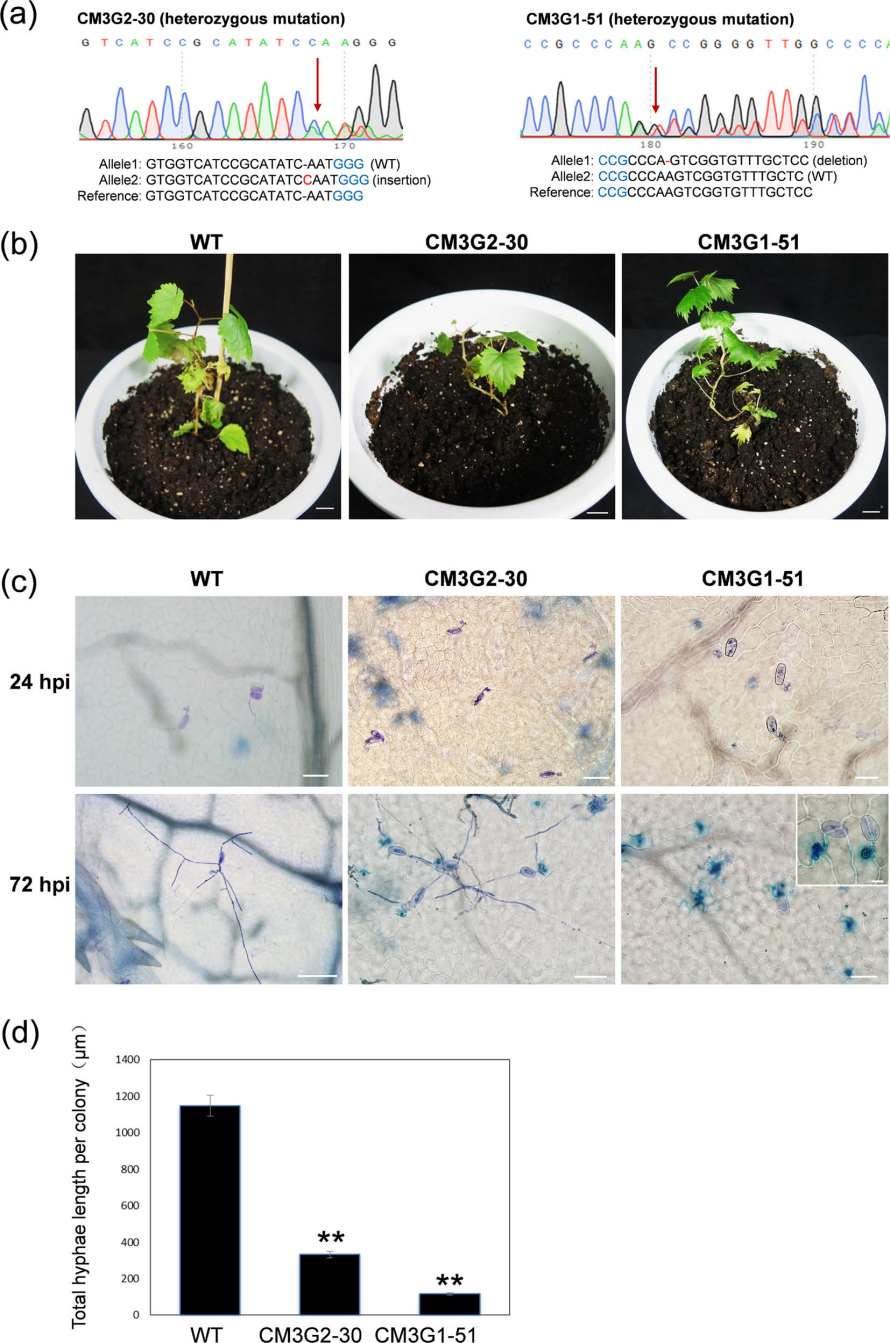
Different types of mutations confer different levels of resistance. **a** Sequence chromatograms of mutation lines CM3G2-30 and CM3G1-51. The first substitution or indel sites are indicated with red arrowheads. The protospacer-adjacent motif (PAM) sequences are highlighted in blue, and the indels or substituted bases are highlighted in red. ‘−’ indicates deletions. **b** Comparison of the wild-type (WT) and different types of *VvMLO3*-edited lines 15 days after transplantation from subculture medium. **c** Representative micrographs showing the growth of powdery mildew in the WT and *VvMLO3*-edited lines at 24 and 72 hpi. Bars = 100 μm except for that (20 μm) in the inset. **d** Total hyphal length per powdery mildew colony on the leaves of the *VvMLO3*-edited lines and WT inoculated with *En* NAFU1 at 72 hpi. Data are the means ± SE calculated from three replicate experiments. Asterisks indicate significant differences between the edited lines and the Thompson Seedless WT control (*P* < 0.01; *n* = 3, Student’s *t* test).

**Fig. 6:**
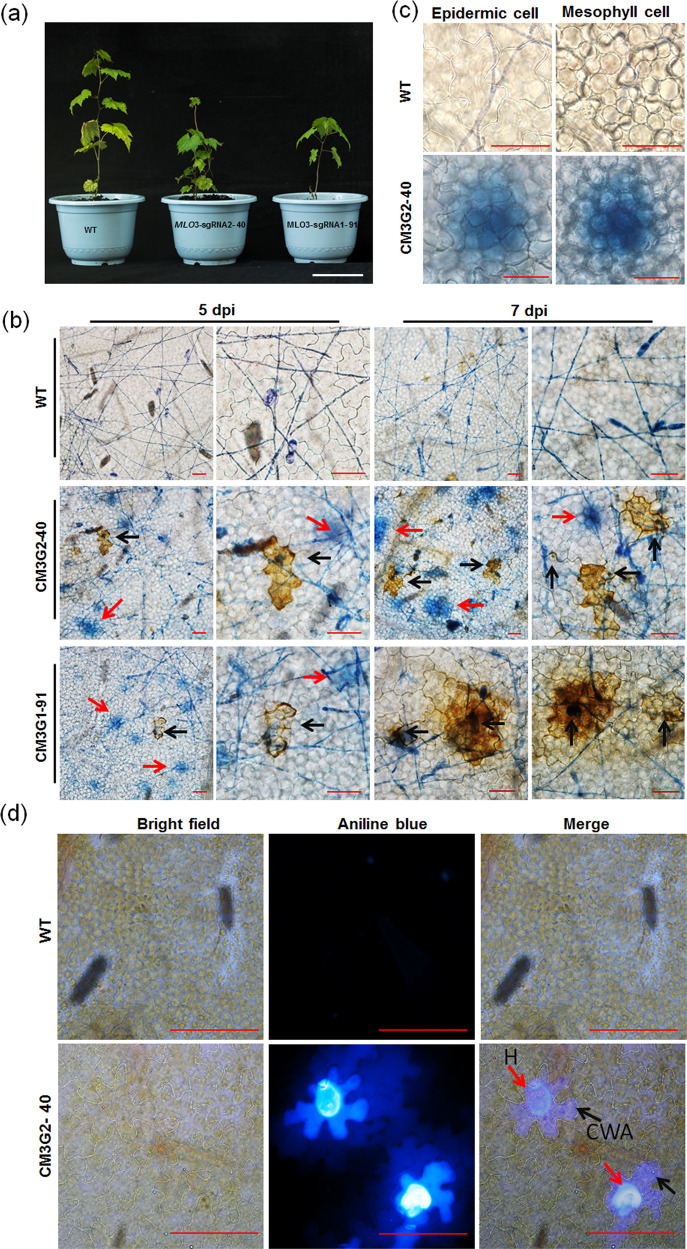
*VvMLO3-*edited grapevine plants show infection-triggered cell death, H_2_O_2_ accumulation and CWAs. **a** The wild-type (WT) and two *VvMLO3-*edited heterozygous mutant grapevine lines grown under phytotron conditions for 6 months (bar = 10 cm). **b** Representative micrographs showing DAB- and trypan blue-stained epidermal cells of the WT and *VvMLO3*-edited lines at 5 or 7 dpi. Red arrowheads indicate trypan blue retention, and black arrowheads indicate H_2_O_2_ accumulation (bar = 50 μm). **c** Representative images showing a trypan blue-stained leaf section of WT or CM3G2-40 with a focus on either the epidermal layer or the mesophyll cell layer at 7 dpi (bars = 50 μm). **d** Histochemical analysis of infection-triggered CWAs of epidermal cells of the WT and the heterozygous CM3G2-40 mutant line at 7 dpi. Red arrowheads indicate haustoria (H), and black arrowheads indicate infection-triggered CWAs (bars = 50 μm).

To evaluate the effect of targeted mutations in the two *VvMLO* genes on resistance to powdery mildew, two *VvMLO3-*edited lines, CM3G2-30 and CM3G1-51 (sequencing chromatograms are shown in Fig. 5a), and one nontransgenic control plant were transplanted to pots (Fig. 5b), and detached leaves of these plants were inoculated with the powdery mildew isolate *En*. NAFU1^33^ 15 days after transplantation. Leaves inoculated with *En*. NAFU1 were collected at 24 and 72 hpi and stained with trypan blue for the visualization of fungal structures by microscopy. At 24 hpi, both the *VvMLO3*-edited and nontransgenic lines produced appressoria with no apparent differences. However, at 72 hpi, the edited line showed much less fungal growth compared with the control line (Fig. 5c), with only ~1/4 of the total hyphal length per colony being observed on CM3G2-30 and ~1/10 on CM3G1-51 compared with that in the control line (*P* < 0.01) (Fig. 5d). In addition, there were fewer secondary hyphae on the two *VvMLO3*-edited lines compared with the nontransgenic control (not shown), indicating that both edited lines exhibited enhanced resistance to powdery mildew. Unfortunately, all five *VvMLO4*-edited lines produced deformed leaves and died. Thus, it remains to be determined whether VvMLO4 plays a role in powdery mildew pathogenesis. The potted *VvMLO3*-edited plants were transferred to a greenhouse for further growth and evaluation (Fig. 6a). In the edited lines harboring heterozygous mutations in *VvMLO3*, clear powdery mildew-induced cell death was observed (Fig. 6b). Careful examination of the trypan blue-stained leaf sections revealed that in many cases, the mesophyll cells underneath the infected epidermal cells were stained blue (Fig. 6c), suggesting that signals from mildew-invaded epidermal cells trigger the collapse of neighboring mesophyll cells as a result of the mutation of *VvMLO3* in these heterozygous lines.

In a previous report, increased cell wall apposition (CWA) or papilla formation was observed beneath the site of attempted penetration in *mlo* mutant plants^21^. We used aniline blue staining to check for the presence of callose in the CWAs after the inoculation of powdery mildew onto the detached leaves of *VvMLO3*-edited lines. At 7 dpi, clear CWAs were observed in the heterozygous mutant line CM3G2-40 but not in nontransgenic control plants (Fig. 6d). In addition, H_2_O_2_ production and accumulation visualized as brownish precipitates upon 3,3′-diaminobenzidine (DAB) staining were more pronounced in mildew-infected cells of the heterozygous mutant lines CM3G1-91 and CM3G2-40 at 7 dpi (Fig. 6b and Fig. S3a). The quantification of H_2_O_2_ accumulation using ZEN 2012 software showed that ~22% and ~42% of the infected leaf epidermal cells of the two *VvMLO*-edited plants exhibited H_2_O_2_ accumulation, which was three to six times higher than that (6.5%) in the infected leaf epidermal cells of the wild-type plants (Fig. S2a, b). Taken together, our data suggest that *Vvmlo3-*mediated resistance to powdery mildew is associated with callose deposition and H_2_O_2_ accumulation in powdery mildew-infected epidermal cells and possibly the demise of mesophyll cells underneath.

**Fig. 7:**
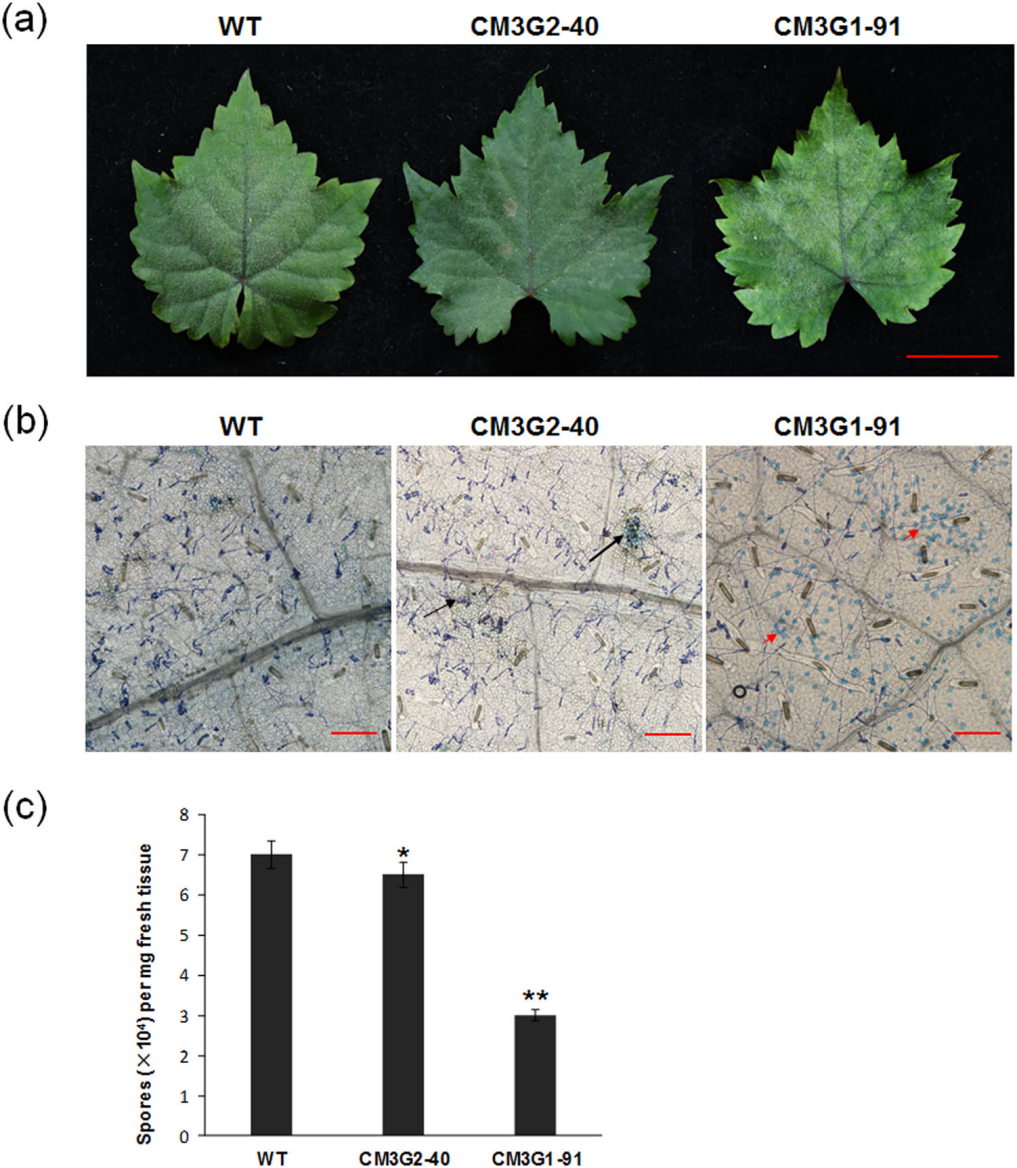
Targeted editing of *VvMLO3* improved the resistance of *V. vinifera* cv. Thompson Seedless to powdery mildew. **a** Representative *En*. NAFU1-infected leaves of the *MLO*-edited lines in comparison with those of the WT control line at 15 dpi (bar = 5 cm). **b** Detection of cell death by trypan blue staining in leaves of *VvMLO3*-edited grapevine at 15 dpi (bars = 50 μm). Black arrows indicate leaf cell necrotic lesions, while red arrows denote clustered dead or dying mesophyll cells stained by trypan blue. **c** Quantification of the number of spores per mg of fresh leaf tissue in WT and *VvMLO3*-edited grapevines at 20 dpi. Each data point is the mean of three independent experiments (±standard deviation); asterisks indicate significant differences in comparison with the WT as determined by Student’s *t* test (*P < 0.05, **P < 0.01).

To assess the impact of the *VvMLO3* gene mutation on whole-plant resistance to powdery mildew, two independent experiments were carried out. Potted transgenic or nontransgenic control plants were inoculated with fresh conidia of powdery mildew *En*. NAFU1. This strain was able to infect both the WT control and heterozygous mutant lines CM3G1-91 and CM3G2-40 (Fig. 7a). However, it was noted that the leaves of the edited lines, but not those of the control line, were covered with abundant white velvety fungus at 15 dpi, which seemed to correlate with the presence of necrotic lesions in the infected leaf tissue in the edited lines but not in the control line at 15 dpi (Fig. 7b). The quantification of the number of spores per mg of fresh infected leaf tissue at 20 dpi revealed that there was an ~2-fold reduction in fungal sporulation in the edited line CM3G1-91 in comparison with the WT line, while a small, yet still statistically significant reduction was detected in the other edited line, CM3G2-40 (Fig. 7c).

## Discussion

Since the first report on the application of CRISPR/Cas9 technology for the editing of grapevine genes in 2016^34^, two more recent studies have explored the use of this new genome-editing technology in grapevine using protoplasts^11^ or embryogenic callus cells^9^. Two other examples of successful targeted mutagenesis using the CRISPR/Cas9 system in grapevine resulted from studies conducted for the functional characterization of the *WRKY52* gene^10^ and the *VvCCD8* gene^35^. In this study, two grapevine *MLO* genes, *VvMLO3* and *VvMLO4*, were edited by using CRISPR/Cas9 technology, and the reduction of *VvMLO3* expression by mutating one allele significantly improved the resistance of a susceptible grapevine cultivar against the powdery mildew isolate *En*. NAFU1.

Charrier et al. performed targeted mutagenesis in apple and pear using the CRISPR-Cas9 system, and they classified the obtained mutants as homozygous, heterozygous, biallelic, and chimeric^36^. According to their classification, in a “homozygous” line, both alleles of the target gene contain the same mutation; in a “heterozygous” line, only one allele is mutated; in a “biallelic” line, both alleles are mutated, but the mutations are not identical; and finally, in a “chimeric” line, more than two alleles are detected, which is indicative of the presence of cell lineages derived from different edited cells during somatic embryogenesis. In this study, all four abovementioned types of grapevine mutants were detected, and the “chimeric” type accounted for 45% of the mutants obtained, suggesting that CRISPR/Cas9 is enzymatically active during the early stage of embryogenesis initiated from a single transformed somatic cell.

As a woody perennial fruit crop, grapevine is known to be recalcitrant to genetic transformation. To increase the chance of success, we designed two sgRNAs for each of the two *MLO* target genes, one of which targeted a site near the initiation codon, while the other targeted a site in the middle of the coding sequence for each gene (Fig. 1a). This strategy was found to be effective for *VvMLO3*, as mutations were identified in two target sites. For *VvMLO4*, sgRNA3 (CM4G3) achieved an ~23% editing efficiency, but sgRNA4 (CM4G4) failed to produce a single mutant line (Table 1). The latter situation may simply be due to the insufficient number (eight) of transgenic lines generated or to the presence of unusual local chromatin structures that are less accessible by CRISPR-Cas9^37^. The DNA mutations induced by CRISPR/Cas9 in plants mainly consist of short indels and replacements that occur during the process of DSB repair via nonhomologous end joining (NHEJ)^38^. Such diverse mutations are expected to occur during NHEJ-mediated DNA repair. This mutation pattern was also observed in this study, as most of the mutations induced in *VvMLO3* were indels. Among the 20 edited lines obtained, only one was homozygous for a single 1-bp insertion, while all other lines showed a combination of different types of mutations (Fig. 3c).

*MLO* homologs have been subjected to targeted mutagenesis in many plants for the engineering of powdery mildew resistance^20,21,27,39,40^. The loss of *AtMLO2*, but not *AtMLO6* or *AtMLO12*, in *Arabidopsis thaliana* enhances the resistance of the mutant plants to powdery mildew, while the loss of all three genes results in complete resistance^21^. These findings indicate that while AtMLO2 is the major host S protein required for powdery mildew infection, AtMLO6 and AtMLO12 possess functions that overlap with those of AtMLO2. In this study, *VvMLO3* and *VvMLO4*, from the clade corresponding to *AtMLO2, 6*, and *12* in the phylogenetic tree^29^ (Fig. S3), were selected for CRISPR/Cas9-mediated mutagenesis. Unfortunately, the only *Vvmlo3* homozygous mutant plantlet in which *VvMLO3* was knocked out died of massive leaf necrosis in the tissue culture medium (data not shown). This implies that either the loss of *VvMLO3* or a genetic mutation due to the T-DNA insertion might have triggered lethal cell death in this *Vvmlo3* mutant. Nevertheless, although multiple *VvMLO3*-edited lines were heterozygous lines, qRT-PCR revealed that there was an ~4-8-fold reduction in the VvMLO3 expression level in the edited lines CM3G1-91 and CM3G2-40 in comparison with the WT line (Fig. S4), which indicated that the VvMLO3 expression level was indeed negatively impacted by such editing. Significantly enhanced mildew resistance was observed in multiple *VvMLO3* heterozygous lines in comparison with the WT plants from 2 to 7 dpi (Figs. 5 and 6), suggesting that VvMLO3 is one of the functional homologs of AtMLO2 in grapevine. Taken together, our results suggest that *VvMLO3* is an important (though not the only) host susceptibility gene required for powdery mildew infection. It should note that the nomenclature for grapevine *MLO* genes differed in two previous studies by Feechan^29^ and Winterhagen^41^ that were published around the same time. In the present study, the naming of *VvMLO3* and *VvMLO4* followed the Feechan nomenclature^29^, whereas these genes were named *VvMLO11(W)* and *VvMLO13(W)*, respectively, according to Winterhagen^41^ (for clarity, when we describe MLO genes using the Winterhagen nomenclature, we use *VvMLO6* (W) and *VvMLO7* (W)) (Fig. S3). Previous studies showed that *VvMLO6* (W) and *VvMLO7* (W), rather than *VvMLO11* (W) or *VvMLO13* (W) (i.e., *VvMLO3* and *VvMLO4* according to the Feechan nomenclature), may negatively regulate disease resistance^29,42^, and the silencing of the *VvMLO6* (W) and *VvMLO7* (W) genes resulted in enhanced resistance to powdery mildew^30^. Hence, it is likely that *VvMLO6/7* (W) may also be a functional homolog of *AtMLO2*. Simultaneous targeted mutagenesis of all three grapevine *MLO* genes may therefore be necessary to develop complete mildew resistance in grapevine in the future.

In addition to powdery mildew resistance, *mlo* mutant plants display premature leaf chlorosis and a reduced grain yield in barley^19^, increased callose deposition in the cell wall of leaves in pathogen-free plants and slower growth and development in *A. thaliana*^21,43,44^, and reduced plant size in pepper^45^. Not surprisingly, similar growth phenotypes, such as early leaf senescence, were observed in our *VvMLO3* heterozygous mutant grapevine plantlets (Fig. 4a). Interestingly, mildew-induced mesophyll cell death was observed in the *VvMLO3* heterozygous mutant lines (Fig. 6), which, along with H_2_O_2_ accumulation and callose deposition in mildew-infected epidermal cells, seemed to be associated with the increased resistance of these mutant lines to powdery mildew (Fig. 6). In this regard, it is worth pointing out that no obvious pleiotropic phenotypes were observed when *VvMLO6, 7, 11*, or *13* (W) was knocked down by RNAi^30^. This implies that *VvMLO6, 7*, and *13* (W) may be functionally distinct from *VvMLO3* and/or that these four genes are functionally redundant or were not adequately silenced.

### Conclusion

CRISPR/Cas9-enabled targeted mutagenesis suggested that *VvMLO3* is an S-gene required for the powdery mildew infection of grapevine and that the targeted mutation of *VvMLO3* in a grapevine cultivar results in enhanced resistance to powdery mildew. This demonstrates that CRISPR/Cas9-targeted mutagenesis can be used to develop disease-resistant cultivars and facilitate the functional characterization of genes of interest in grapevine.

## Materials and methods

### Plant materials

*V. vinifera* cv. Thompson Seedless was used for gene cloning and transformation and as a control in gene functional analysis and phenotypic observations of powdery mildew infections. Plants were grown in a phytotron at Northwest A&F University, Yangling, Shaanxi, People’s Republic of China. The temperature was controlled between 22 and 26 °C under a 14 h/10 h (day/night) light cycle. Embryogenic calli were induced from anther filaments of Thompson Seedless. Proembryogenic masses (PEMs) and somatic embryos of Thompson Seedless at the mid-cotyledonary stage of development were used for transformation. PEM cultures were maintained on MS basal medium supplemented with 60 g·L^−1^ sucrose, 3 g·L^−1^ Phytagel, and 1 g·L^−1^ activated charcoal with a pH of 5.8–6.0. PEM cultures were reinduced by placing chopped torpedo-shaped-stage or mid-cotyledonary-stage embryos on KBN medium (MS, sucrose, 1 g·L^−1^ myo-inositol, 0.3 g·L^−1^ KNO_3_, 1.126 mg·L^−1^ 6-BA, 0.552 mg·L^−1^ 2,4-D and 0.505 mg·L^−1^ NOA). The secondary regeneration of PEMs was typically observed within 3–7 months. All cultures were maintained in the dark at 25 °C and transferred to fresh media monthly^46^.

### Cloning of grapevine *MLO* genes

Total RNA was extracted from Thompson Seedless leaves using the E.Z.N.A. Plant RNA Kit (Omega, Guangzhou, China) according to the manufacturer’s instructions. First-strand cDNA was generated from 1.5 μg of total RNA using PrimeScript RTase (Takara Bio Inc., Dalian, China). The synthesized cDNA product was diluted 10-fold and used as a template for the cloning of *MLO* genes. Two *VvMLO* genes, *VvMLO3* and *VvMLO4*, identified in a previous study^29^ were selectively amplified using oligonucleotide primers derived from *VvMLO* gene sequences available in the National Center for Biotechnology Information (NCBI) database (https://blast.ncbi.nlm.nih.gov/Blast.cgi). PrimeSTAR HS DNA Polymerase was used for amplification. The PCR products were cloned into the pMD18-T vector (Takara Bio Inc., Dalian, China) and sequenced at the Beijing AuGCT Biotech, Yangling, Sequencing Department. The sequenced DNAs were aligned with the genome of *Vitis vinifera* PN40024.

### CRISPR-Cas9 binary construct design

Target sequences were selected within two *VvMLO* exons, and four sgRNAs, designated sgRNA1 to sgRNA4, were designed within the two coding regions (Table S3). To increase the editing success rate for each gene, we selected two target loci in each *MLO* gene, seeking loci with a moderate GC content. We screened the target sequences to minimize potential off-target alignments using the online software Grape-CRISPR (http://biodb.sdau.edu.cn/gc/) and CRISPR-P (http://crispr.hzau.edu.cn/CRISPR2/)^47^. For each gene, we located one target site near the start codon and another in a region corresponding to the functional domain. Next, we performed a BLAST-P search based on the National Center for Biotechnology Information (NCBI) database (https://blast.ncbi.nlm.nih.gov/Blast.cgi) of the target sequences (including PAM) against the grapevine genome sequence to ensure that the target sequence was as specific as possible. We avoided sequences containing more than four consecutive Ts to prevent RNA Pol III from using such sites as a transcription termination signal. (The selected target sequences are shown in Fig. 1a.) Target adapter oligos were synthesized for each target locus using Ao ke ding sheng (http://www.augct.com/?ClassID=2) in the Yangling department (Table S2).

The binary pYLCRIPSR/Cas9-N^48^ vector and two sgRNA cassettes driven by the *AtU3b* and *AtU6-1* promoters were used to generate vectors expressing CRISPR/Cas9-*MLO*s. For each sgRNA, a pair of DNA oligonucleotides was synthesized by Beijing AuGCT Biotech and annealed to generate dimers, which were subsequently integrated upstream of the sgRNA scaffolds in the plasmid vector. Two sequential rounds of overlapping PCR were carried out to insert double-stranded, gRNA spacer DNA sequences between double *Bsa* I restriction enzyme sites to generate intermediate vectors with one or two target sites. Intermediate sgRNA expression cassettes with target sequences were generated by overlapping PCR (Figs. S5 and S6). We used a binary CRISPR/Cas9 vector, pYLCRISPR/Cas9P35S-N containing the CaMV 35S promoter and the NPTII gene as a selectable marker. The Cas9 vector was built in the backbone of pCAMBIA1300. The final purified PCR products were assembled into the binary pYLCRIPSR/Cas9 vector containing the *NPT*II gene and 2 × 35 s promoter by Golden Gate ligation^49^. The ligated products with one or two sgRNA expression cassettes were directly used to transform DH5α *E. coli* competent cells.

### *Agrobacterium*-mediated transformation of grapevine

Transformation was carried out via the *Agrobacterium*-mediated transformation system as previously described^50^ with minor modifications. *Agrobacterium tumefaciens* strain GV 3101 was used for transformation. Transformation was carried out in several batches to avoid failure. The bacterial solution (OD600, 0.6–0.8) was poured into a sterile flask, and the grapevine tissue was soaked in the flask for 15–20 min with gentle shaking. The tissue was then placed on two layers of sterile filter paper to remove excess bacterial culture and transferred to 2 ml of liquid MS medium (containing 20 mg·L^−1^ AS and 3% sucrose) for cocultivation. After 2 days of cocultivation at 25 °C in the dark, the material was further maintained for 1 month without any antibiotics before screening to allow the transformed material to recover. The tissue was then washed three times in sterile water and transferred to KBN delayed-screening medium (30 g·L^−1^ sucrose, MS, 0.3 g·L^−1^ KNO_3_, 1.126 mg·L^−1^ 6-BA, 1 g·L^−1^ myo-inositol, 0.505 mg·L^−1^ NOA, Phytagel, 200 mg·L^−1^ Cef) for 3 weeks, followed by incubation for 1 week on X3 delayed-screening medium (30 g·L^−1^ sucrose, MS, 1 g·L^−1^ activated charcoal, Phytagel, 200 mg·L^−1^ Cef). After this delayed screening, the transformed PEM cultures were transferred to resistance screening medium containing Cef and Kan (30 g·L^−1^ sucrose, MS, 1 g·L^−1^ activated charcoal, Phytagel, 200 mg·L^−1^ Cef, 75 mg·L^−1^ Kan). Screening was continued until germinated embryos developed. Germinated resistant embryos were then transferred to germination medium (15 g·L^−1^ sucrose, MS, Phytagel) and placed under light for further development. After true leaves appeared, the cultured plantlets were transferred to 30 mL rooting medium (30 g·L^−1^ sucrose, MS, 1 g·L^−1^ activated carbon, Phytagel, 1 mg·L^−1^ IBA) for further development. Rooted plantlets were transplanted into pots (diameter: 14 cm, height: 10 cm) containing potting mix (perlite, vermiculite, and peat) and covered with a transparent plastic cup, then placed in a phytotron.

### Genotyping and mutant verification

The genotyping of transgenic lines was carried out essentially as described previously^31^. The genomic DNA of each line was isolated from 0.5 to 1.0 g of regenerated plantlet tissue using a CTAB-based method^51^. To verify the presence of the engineered sequences, specific oligonucleotide primers (SP-L-35S and SP-R) were used to amplify the CRISPR/Cas9 sequence (Table S2). The PCR products (ca. 400–600 bp) were sequenced directly using internal specific primers, for which the ideal binding positions are ~150–250 bp upstream of the target sites (Beijing AuGCT Biotech Yangling Sequencing Department). The detection of target gene editing requires the sequencing of multiple clones of the PCR amplicons derived from the same genomic DNA sample. The PCR amplicons were cloned into a pMD18-T plasmid vector, and five to ten randomly selected *E. coli* colonies were used for plasmid preparation and insert sequencing. SnapGene Viewer software was used for mutation analysis. The sequence results are shown in Table S1.

### Pathogen inoculation and evaluation of resistance to powdery mildew

Four *MLO*-edited lines, CM3G1-51 and -91 and CM3G2-30 and -40, were assessed for resistance to powdery mildew. The transgenic mutant and nontransgenic control lines were inoculated with the powdery mildew isolate *En* NAFU1 as described previously^33^. For detached leaf inoculation, CM3G2-30, CM3G1-51, and wild-type (WT) plants were used at 15 days after transplantation from the subculture medium. Fully expanded leaves at the third position from the shoot tip were chosen for inoculation. Representative micrographs and hyphal development were observed at 24 and 72 hpi, respectively. For plant leaf inoculation, CM3G2-40, CM3G1-91, and wild-type (WT) plants were grown under phytotron conditions for 6 months for propagation and then subjected to inoculation. DAB and trypan blue-stained epidermal cells of WT, CM3G2-40 and CM3G1-91 plants were observed at 5 or 7 dpi. To assess the impact of the *VvMLO3* gene mutation on whole-plant resistance to powdery mildew, plants of the transgenic lines CM3G2-40 and CM3G1-91 or nontransgenic control plants that had been grown in pots for six months were inoculated with fresh conidia of *En*. NAFU1 powdery mildew, and the detection of cell death was performed by trypan blue staining in the leaves of CM3G2-40 or CM3G1-91 plants at 15 dpi. Three individuals from each CM3G2-40 and CM3G1-91 line were evaluated. Symptoms of powdery mildew infection were evaluated after inoculation using an Olympus BX51 microscope under visible light. DAB staining, trypan blue staining and aniline blue staining were carried out as previously described to detect the accumulation of H_2_O_2_, fungal structures, cell death and CWAs^52^.

### Statistical analysis

Disease severity data were analyzed with ZEN 2012 and SigmaPlot 10.0 software. All data were analyzed using paired Student’s *t*-tests (http://www.physics.csbsju.edu/stats/). The mean values ± standard deviation of the mean (SD) were calculated based on the results of at least three replicates, and significant differences compared with controls are represented by *p < 0.05 and **p < 0.01.

## Supplementary information

Supporting tables

Supporting figures
