# Supplementary material for: CRISPR/Cas9-mediated mutagenesis of VvMLO3 results in enhanced resistance to powdery mildew in grapevine (Vitis vinifera)
Source: Hortic Res. 2020 Aug 1;7:116. doi: 10.1038/s41438-020-0339-8 (PMC7395163; doi:10.1038/s41438-020-0339-8)
Supplement: Supplementary file 1 — Supporting tables [file 41438_2020_339_MOESM1_ESM.doc]

Table S1 Nucleotide sequence and mutation type of several targeted genomic mutations

| Gene: CM3G1; Target sequence: CCGCCCAAGTCGGTGTTTGCTCC  Heterozygous: 1; Biallelic: 2; Homozygous: 0；chimaric: 3 | | |
| --- | --- | --- |
| CM3G1-1 | CCGCCC- - -TCGGTGTTTGCTCC  CCGCCC- -GTCGGTGTTTGCTCC  CCGCCCAAGTCGGTGTTTGCTCC | chimaric |
| CM3G1-5 | CCGCCCCACCGAAGATGGATC  CCGCCCAAAGTCGGTGTTTGCTCC | biallelic |
| CM3G1-7 | CCGCCC- -GTCGGTGTTTGCTCC  CCGCCCAAAGTCGGTGTTTGCTCC  CCGCCC- AGTCGGTGTTTGCTCC  CCGCCCTAAGTCGGTGTTTGCTCC | chimaric |
| CM3G1-19 | CCGCCCAAAGTCGGTGTTTGCTCC  CCGCCC- -GTCGGTGTTTGCTCC  CCGCCCC- - - - -GGTGTTTGCTCC | chimaric |
| CM3G1-25 | CCGCCC- -GTCGGTGTTTGCTCC  CCGCCC- -GTCGGTGTTTGCTCC | biallelic |
| CM3G1-51 | CCGCCC- AGTCGGTGTTTGCTCC  CCGCCCAAGTCGGTGTTTGCTCC | heterozygous |

| Gene: CM3G2; Target sequence: GTGGTCATCCGCATATCAATGGG  Heterozygous: 3; Biallelic: 0; Homozygous: 1；chimaric: 0 | | |
| --- | --- | --- |
| CM3G2-12 | GTGGTCATCCGCATATCAAATGGG | homozygous |
| CM3G2-25 | GTGGTCATCCGCATATCAAATGGG  GTGGTCATCCGCATATCAATGGG | heterozygous |
| CM3G2-30 | GTGGTCATCCGCATATCCAATGGG  GTGGTCATCCGCATATCAATGGG | heterozygous |
| CM3G2-40 | GTGGTCATCCGCATATCCAATGGG  GTGGTCATCCGCATATCAATGGG | heterozygous |

Table S2 The primer used in this study

| Primer Names | Sequence (5’-3’) | Description |
| --- | --- | --- |
| *MLO*4F | AAAATGGCTGGGGCAACCGGAGGAAG | gene amplification |
| *MLO*4R | TTTCGTCCTCTCTCTTCGATCAAATGA | gene amplification |
| *MLO*3F | AAAATGGCTAAGGGATCAAAGGATCG | gene amplification |
| *MLO*3R | TTTAGTTCTTGCTCTTTTATCGAATGA | gene amplification |
| U-F | CTCCGTTTTACCTGTGGAATCG | vector construction |
| gRNA-R | CGGAGGAAAATTCCATCCAC | vector construction |
| M4gRT3#+ | CTGCTCCAGCGACCTTCCTCgttttagagctagaaat | vector construction |
| M4gRT4#+ | CTTTCAGAACGCATTTCAGCgttttagagctagaaat | vector construction |
| M3gRT1#+ | GAGCAAACACCGACTTGGGgttttagagctagaaat | vector construction |
| M3gRT2#+ | TGGTCATCCGCATATCAATgttttagagctagaaat | vector construction |
| M4AtU3bT3#- | GAGGAAGGTCGCTGGAGCAGTgaccaatgttgctcc | vector construction |
| M4AtU3bT4#- | GCTGAAATGCGTTCTGAAAGTgaccaatgttgctcc | vector construction |
| M3AtU6-1T1#- | CCCAAGTCGGTGTTTGCTCCaatcactacttcgtct | vector construction |
| M3AtU6-1T2#- | ATTGATATGCGGATGACCACaatcactacttcgtct | vector construction |
| Ugccg-B1' | ATAAATTggtctcactcgTGGAATCGGCAGCAAAGG | vector construction |
| gRctga-B2 | ATAATTTggtctcttcagCCATCCACTCCAAGCTC | vector construction |
| Uctga-B2' | ATAAATTggtctcactgaTGGAATCGGCAGCAAAGG | vector construction |
| gRgttt-BR | ATAATTTggtctctaccgCCATCCACTCCAAGCTC | vector construction |
| M4U3bT3F | GTCACTGCTCCAGCGACCTTCCTC | target adapter |
| M4U3bT3R | AAACGAGGAAGGTCGCTGGAGCAG | target adapter |
| M4U3bT4F | GTCACTTTCAGAACGCATTTCAGC | target adapter |
| M4U3bT4R | AAACGCTGAAATGCGTTCTGAAAG | target adapter |
| M3U6-1T1F | ATTGGAGCAAACACCGACTTGGG | target adapter |
| M3U6-1T1R | AAACCCCAAGTCGGTGTTTGCTC | target adapter |
| M3U6-1T2F | ATTGTGGTCATCCGCATATCAAT | target adapter |
| M3U6-1T2R | AAACATTGATATGCGGATGACCA | target adapter |
| SP-L-Ubi | GCGGTGTCATCTATGTTACTAG | positive detection |
| SP-L-35S | GTCGTGCTCCACATGTTGACCG | positive detection |
| SP-R | CCGACATAGATGCAATAACTTC | positive detection |
| mutantM4T3F | AGTCATCCCCATCTTCCCATCCTC | mutation detection |
| mutantM4T3R | GATGCCAAGTTGCTGCAACACTC | mutation detection |
| mutantM4T3-sequencing | GATCATCGCGAGATGGGTGCA | mutation detection |
| mutantM4T4F | GATTGTAAGCGTCGCCTCCATG | mutation detection |
| mutantM4T4R | GTCACCAGGGCATAGAGCGGG | mutation detection |
| mutantM4T4-sequencing | GAGAGGTTGTGAAGGGAGTGCCG | mutation detection |
| mutantM3T1F | GCACAATCATTTCGAGTGACACAA | mutation detection |
| mutantM3T1R | AGGAGCAGCGATATGAACCCC | mutation detection |
| mutantM3T1-sequencing | CGTTTGTTTCTCTTTGTTAGCCACTG | mutation detection |
| mutantM3T2F | GTGGAGCCTGGTGATGATCTTTTCT | mutation detection |
| mutantM3T2R | TGGGCTTCATGGTAGATCCCATC | mutation detection |
| mutantM3T2-sequencing | GAACGCATTCCAGGTTGCCTTCT | mutation detection |

Table S3 Target selection

| Target site(5’-3’) | Naming | Genes |
| --- | --- | --- |
| GGAGCAAACACCGACTTGGGCGG | sgRNA1 | *MLO*3 |
| GTGGTCATCCGCATATCAATGGG | sgRNA2 | *MLO*3 |
| CCGGAGGAAGGTCGCTGGAGCAG | sgRNA3 | *MLO*4 |
| CTTTCAGAACGCATTTCAGCTGG | sgRNA4 | *MLO*4 |
