# Supplementary material for: CRISPR/Cas9-mediated mutagenesis of VvMLO3 results in enhanced resistance to powdery mildew in grapevine (Vitis vinifera)
Source: Hortic Res. 2020 Aug 1;7:116. doi: 10.1038/s41438-020-0339-8 (PMC7395163; doi:10.1038/s41438-020-0339-8)
Supplement: Supplementary file 2 — Supporting figures [file 41438_2020_339_MOESM2_ESM.doc]

Fig. S1


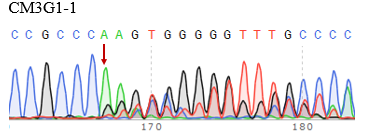

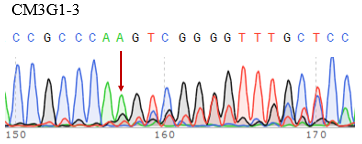


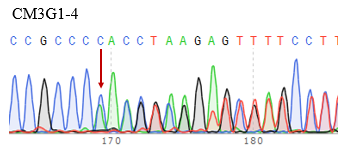

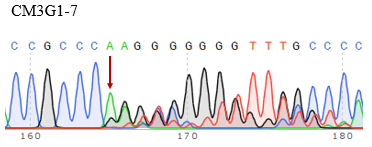


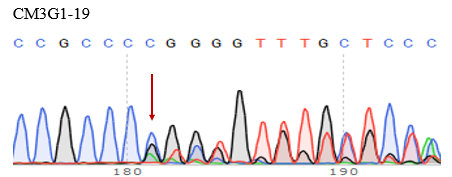

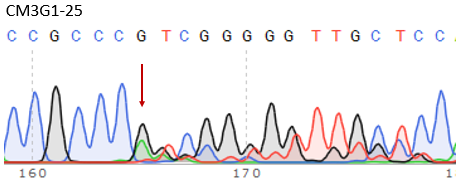

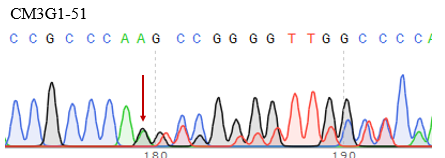

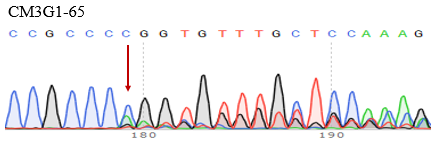

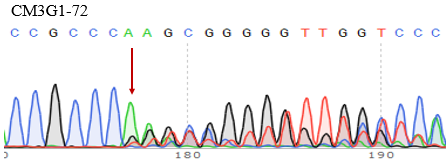

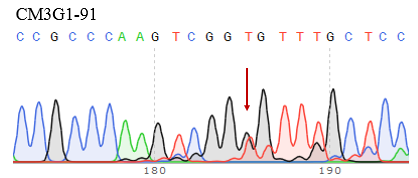


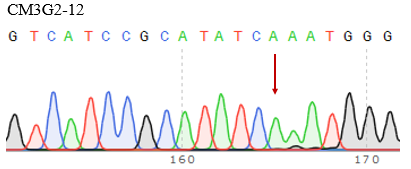

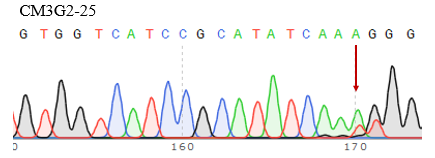

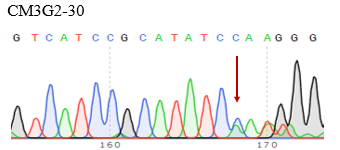

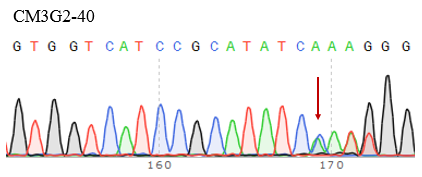


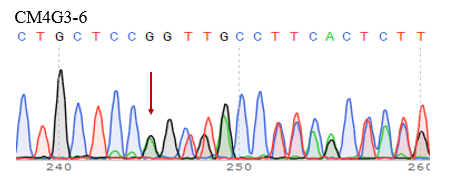

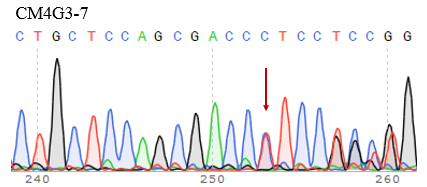


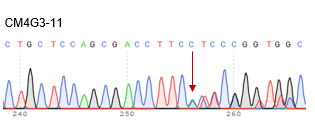

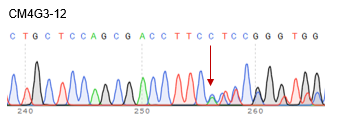


**Fig. S1. Sequence chromatograms of edited lines.** Examples of direct sequencing of PCR products containing targeted sites in edited grapevine lines.

**
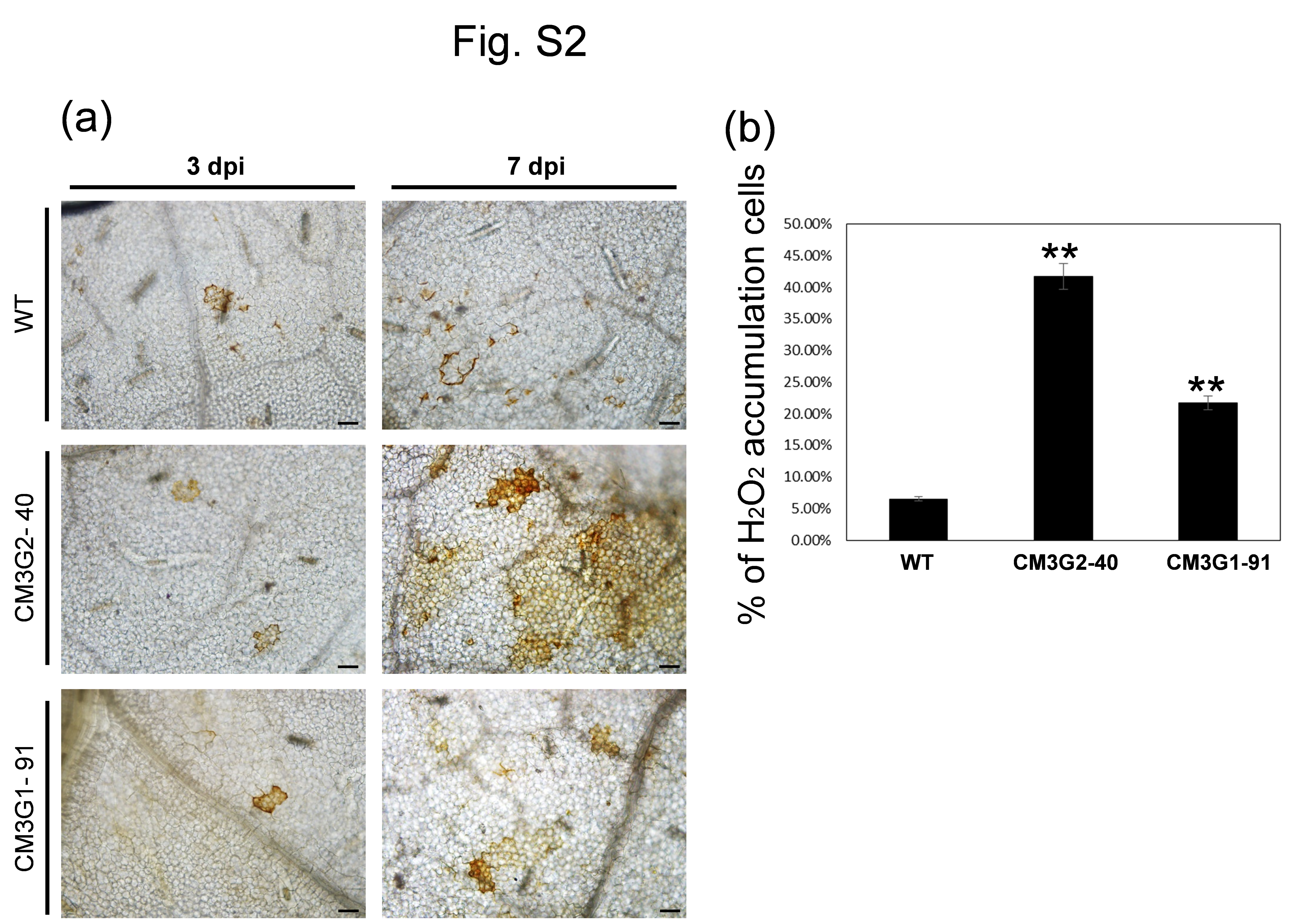
**

**Fig. S2.** ***VvMLO3-*editied grapevine lines show enhanced accumulation of H_2_O_2_ in penetrated epidermal cells.** (a) Enhanced accumulation of H_2_O_2_ as revealed by 3, 3-Diaminobenzidine tetrahydrochloride (DAB) staining of the wild type (WT) and two *VvMLO3* heterozygous mutants at 7dpi (Bars=50 μm). (b) Frequencies of the cells with H_2_O_2_ accumulation at 7 dpi. Data are means ± SE, calculated from three duplicated experiments. ** indicate values highly significantly different from that of the WT ‘Thompson Seedless’ (P < 0.01; n = 3, Student’s *t* test).

Fig. S3


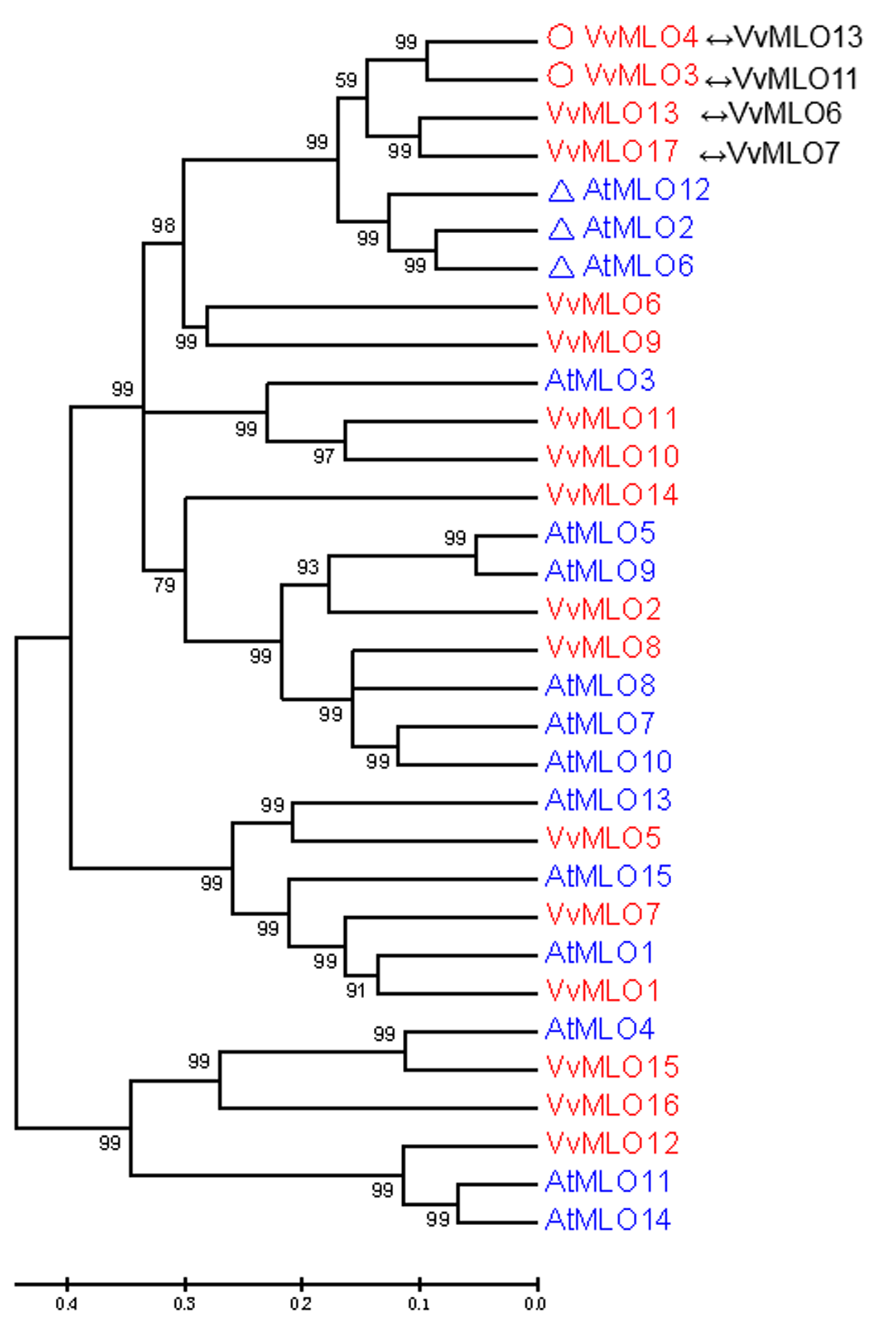


**Fig. S3. Phylogenetic analysis of the grapevine *MLO* genes.** The full-length amino acid sequences of *MLO* genes from *Vitis vinifera* (*Vv*) and *Arabidopsis thaliana* (*At*) were aligned using ClustalX, and the phylogenetic tree was constructed using the neighbor-joining method with 1000 bootstrap replicates with MEGA 5.0. Note, the *VvMLOs* genes (in red) named by Feechan (2008) were used for the analysis. The four genes discussed in the present study are also labeled with their corresponding names (in black) by Winterhagen (2008).

Fig. S4


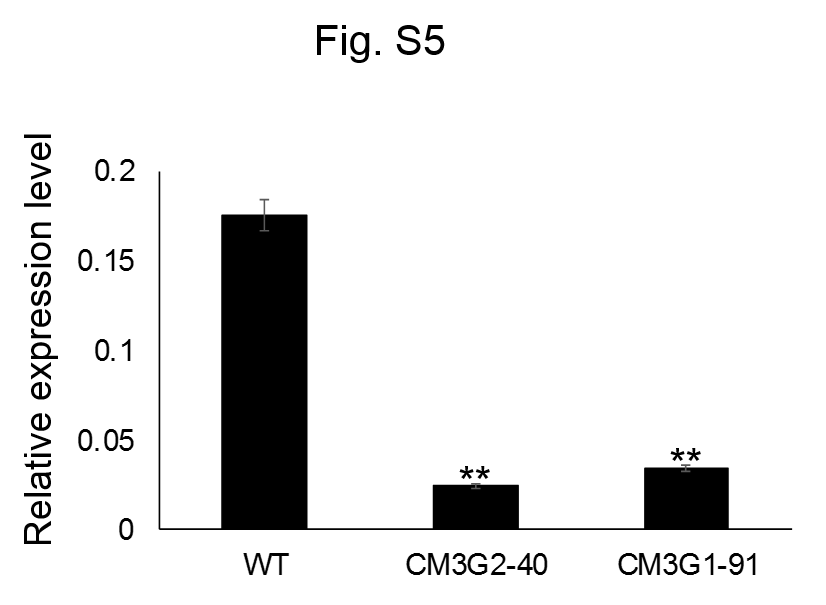


**Fig. S4.** *MLO3* expression levels of three lines WT, CM3G2-40 and CM3G1-91 by qRT-PCR. Actin was used as an internal control. The experiments were repeated three times and provided consistent results. Mean values and SDs were obtained from three biological and three technical replicates. Detailed expression levels of two *VvMLO3*-edited lines that were significantly downregulated. The data represent the mean value ± SD. * and ** represent statistically significant (p < 0.05) and highly significant (p < 0.01), respectively.

Fig. S5


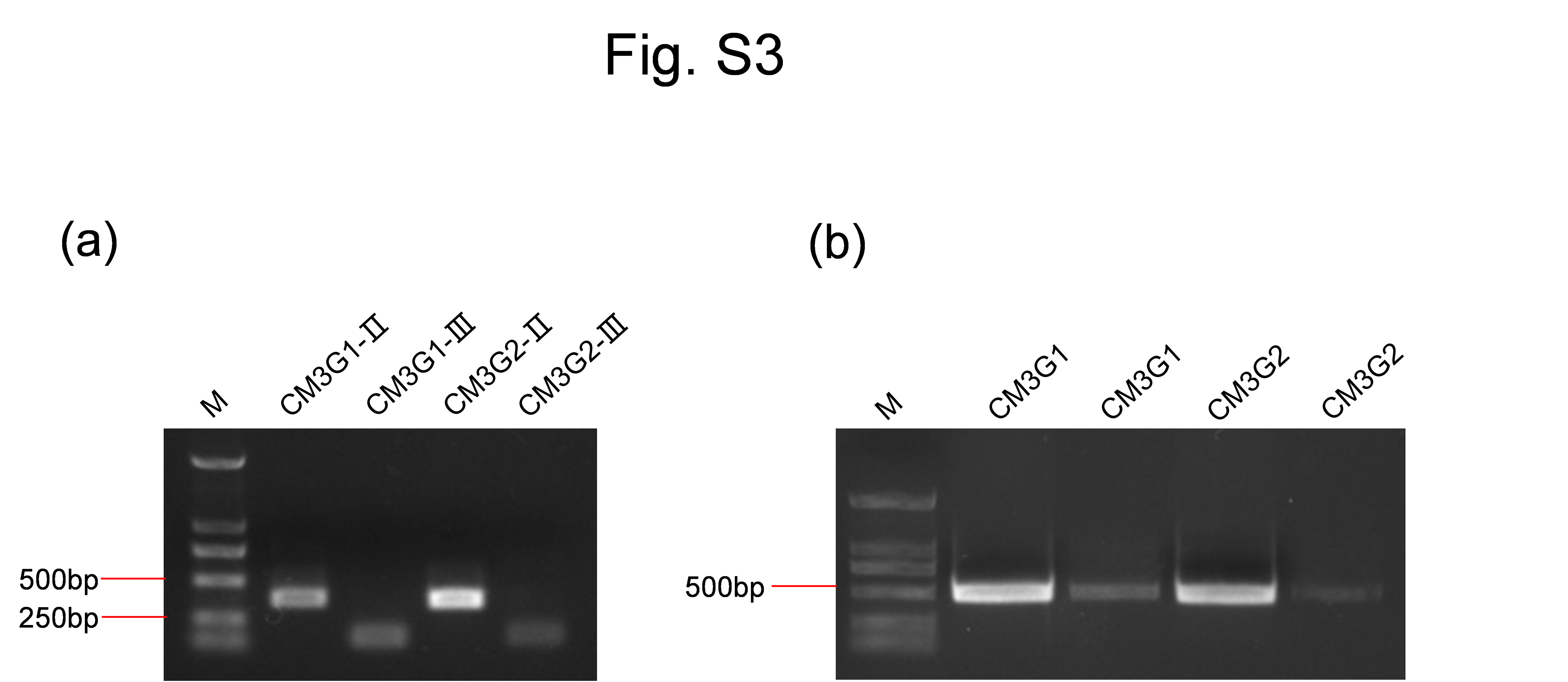


**Fig. S5. Amplification of the sgRNA cassette by overlapping PCR.** (a) Gel image showing DNA fragments amplified by the first-round PCR. (b) Gel image showing DNA fragments amplified by the second-round PCR, M, DS 2000 marker.

Fig. S6

**
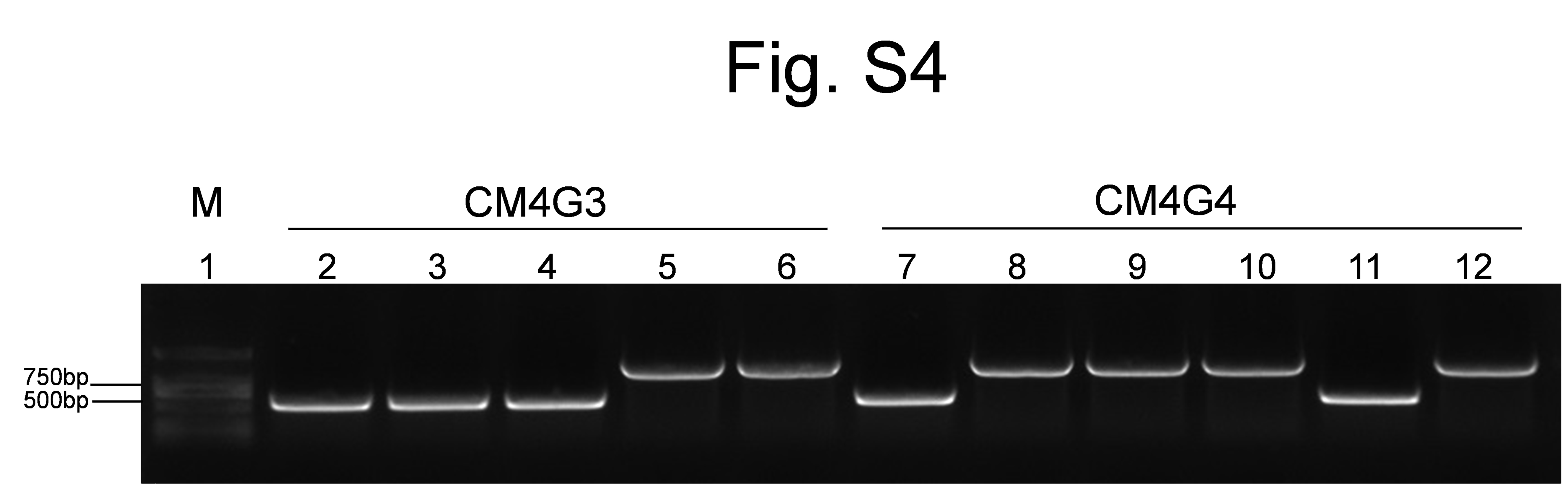
**

**Fig. S6. PCR detection of the mutations in individual clones of the amplicons from the target regions of the two *VvMLO* genes.** Lanes 2, 3 and 4 are positive monoclonal of CM4G3，Lanes 7 and 11 are positive monoclonal of CM4G4. M, DS 2000 marker.
